# Long-term effects of antimicrobial drugs on the composition of the human gut microbiota

**DOI:** 10.1080/19490976.2020.1791677

**Published:** 2020-09-29

**Authors:** M. Mulder, D. Radjabzadeh, J. C. Kiefte-de Jong, A. G. Uitterlinden, R. Kraaij, B. H. Stricker, A. Verbon

**Affiliations:** a Department of Epidemiology, Erasmus MC, Rotterdam, The Netherlands; b Youth and Healthcare Inspectorate, Heerlen, The Netherlands; c Department of Internal Medicine, Erasmus MC, Rotterdam, The Netherlands; d Department of Public Health and Primary Care, LUMC, The Hague, The Netherlands; e Department of Medical Microbiology and Infectious Diseases, Erasmus MC, Rotterdam, The Netherlands

**Keywords:** Gut microbiota, antimicrobial use, macrolides and lincosamides, quinolones, beta-lactams, sulfonamides and trimethoprim, nitrofuran derivatives, tetracyclines

## Abstract

**Introduction:**

Antimicrobial drugs are known to have effects on the human gut microbiota. We studied the long-term temporal relationship between several antimicrobial drug groups and the composition of the human gut microbiota determined in feces samples.

**Methods:**

Feces samples were obtained from a community-dwelling cohort of middle-aged and elderly individuals (Rotterdam Study). Bacterial DNA was isolated and sequenced using V3/V4 16 S ribosomal RNA sequencing (Illumina MiSeq). The time between the last prescription of several antimicrobial drug groups and the day of sampling was categorized into 0–12, 12–24, 24–48 and >48 months. The effects of the antimicrobial drug groups on the Shannon alpha-diversity (diversity), the Bray–Curtis beta-diversity (community structure), the Firmicutes/Bacteroidetes (F/B) ratio and individual genera were determined.

**Results:**

We studied the gut microbiota of 1413 individuals (57.5% female, median age 62.6 years). The alpha-diversity was significantly lower up to 4 years after prescriptions of macrolides and lincosamides. It was also lower in the first year after the use of beta-lactams. The community structure (beta-diversity) of the microbiota was significantly different up to 4 years for macrolides and lincosamides, the first year for beta-lactams and at least the first year for quinolones. For the F/B ratio, drugs with a high anaerobic activity shifted the ratio toward Firmicutes in the first year whereas other antimicrobial drugs shifted the ratio toward Bacteroidetes.

**Conclusion:**

Use of antimicrobial drugs is associated with a shift in the composition of the gut microbiota.These effects differ in strength and duration, depending on the antimicrobial drug group used. These findings should be considered when prescribing antimicrobial drugs.

## Introduction

The gut microbiota plays a role in a variety of processes, such as protection against overgrowth of pathogenic micro-organisms, in the development of the host immune response, in neurologic signaling and in the synthesis and metabolism of several compounds, such as short-chain fatty acids (SCFAs).^1,2^ In particular, the SCFA butyrate is said to have an important function in the maintenance of a healthy colonic epithelium.^3^

The composition of the gut microbiota may differ with age,^4^ gender and BMI^5^ and can change under the influence of diet,^6,7^ physical activity,^8^ diabetes^9^ and use of drugs, such as proton pump inhibitors,^10^ corticosteroids^11^ and statins.^12^ Furthermore, it is known that it can be influenced by the use of antimicrobial drugs (post-antibiotic dysbiosis). The use of antimicrobial drugs has been reported to increase the vulnerability to overgrowth of potentially pathogenic bacteria, such as *Clostridium difficile*, with the risk of pseudomembranous colitis. Moreover, it has been described to cause a loss of diversity of the gut microbiota, cause a decrease of important taxa, alter gene expression, select for intrinsically resistant bacteria, and select for new mutations.^13–15^ Additionally, dysbiosis has also been designated as a factor that promotes horizontal gene transfer, thereby increasing the probability of spreading antibiotic resistance genes.^16^

In adults, some information is available about the effects of specific antimicrobial drug groups on the composition of the gut microbiota. Short-term exposure to clindamycin was shown to cause a shift of the gut microbiota, for example a decline in the diversity of Bacteroidetes.^17^ Furthermore, *Lachnospiraceae* abundancy in the gut was decreased up to 6 months after the use of amoxicillin or azithromycin.^18^ Also, one study showed the effects of using beta-lactam antibiotics in the 12 months before sampling in a population-based cohort.^19^ However, most studies have investigated these effects in small populations, studying rather short-term effects and using a variety of methods to investigate the microbiota. Therefore, the objective of this study was to describe and compare the effects and the duration of the effects of different antimicrobial drug groups on the composition of the human microbiota in feces samples from a large population of community-dwelling middle-aged and elderly individuals using different outcomes that characterize the microbiota.

## Results

We obtained a dataset with microbiota data from 1427 participants, of whom 14 (1.0%) were excluded, because no pharmacy data were available. From the remaining 1413 participants, 812 (57.5%) were female and 601 (42.5%) were male with a median age of 62.6 years (IQR 58.6–66.1), a median BMI of 26.8 (IQR 24.5–29.7) and the feces sample had been in the mail for a median time of 1 day (IQR 1–2 days). Furthermore, 323 individuals used proton pump inhibitors and 252 used a statin. There was no use of tacrolimus and the use of antineoplastic agents (3 participants, 0.2%) was very low; therefore, these drugs were not included in the models. A total of 1281 (90.6%) participants had received at least 1 prescription of an antimicrobial drug during follow-up (at least 17 years). Most participants (73.7%) had 1 or more prescriptions of beta-lactam antibiotics, other frequently used antibacterial drugs were tetracyclines (57.7%) and macrolides and lincosamides (44.0%). The number of prescriptions for amphenicols (J01B), other beta-lactam antibacterials (J01D), aminoglycoside antibacterials (J01G), glycopeptide antibacterials (J01XA), polymyxins (J01XB), steroid antibacterials (J01XC), imidazole derivatives (J01XD) and other antibacterials (J01XX) was very low and these groups could not be analyzed separately (Table 1). The correlations between the different antimicrobial drug groups were low (Table S1).

**Table 1.** Use of antimicrobial drugs in the study population.

Antimicrobial drug group	0–12 months^*^	12–24 months	24–48 months	>48 months	None
Antibacterial for systemic use (J01)	355	217	236	473	132
Tetracyclines (J01A)	100	103	152	461	597
Amphenicols (J01B)	0	0	0	0	1413
Beta-lactam antibacterials (J01C)	179	131	163	569	371
Other beta-lactam antibacterials (J01D)	1	2	1	11	1398
Sulfonamides and trimethoprim (J01E)	20	23	55	267	1048
Macrolides, lincosamides and streptogramins (J01F)	62	49	106	405	791
Aminoglycoside antibacterials (J01 G)	1	0	0	1	1411
Quinolone antibacterials (J01 M)	35	38	48	154	1138
Glycopeptide antibacterials (J01XA)	0	0	0	1	1412
Polymyxins (J01XB)	0	0	0	0	1413
Steroid antibacterials (J01XC)	0	0	0	0	1413
Imidazole derivatives (J01XD)	0	0	0	2	1411
Nitrofuran derivatives (J01XE)	76	38	67	121	1111
Other antibacterials (J01XX)	13	3	5	3	1389

Use of antibacterial drugs per group and overall for each time period of prescription to fecal sampling (0–12, 12–24, 24–48, >48 months, or no use). The use of amphenicols (J01B), other beta-lactam antibacterials (J01D), aminoglycoside antibacterials (J01G), glycopeptide antibacterials (J01XA), polymyxins (J01XB), steroid antibacterials (J01XC), imidazole derivatives (J01XD) and other antibacterials (J01XX) is too low to analyze further.

*Time period between prescription of antimicrobial drug and fecal sampling.

### Shannon alpha-diversity

**Figure 1.**
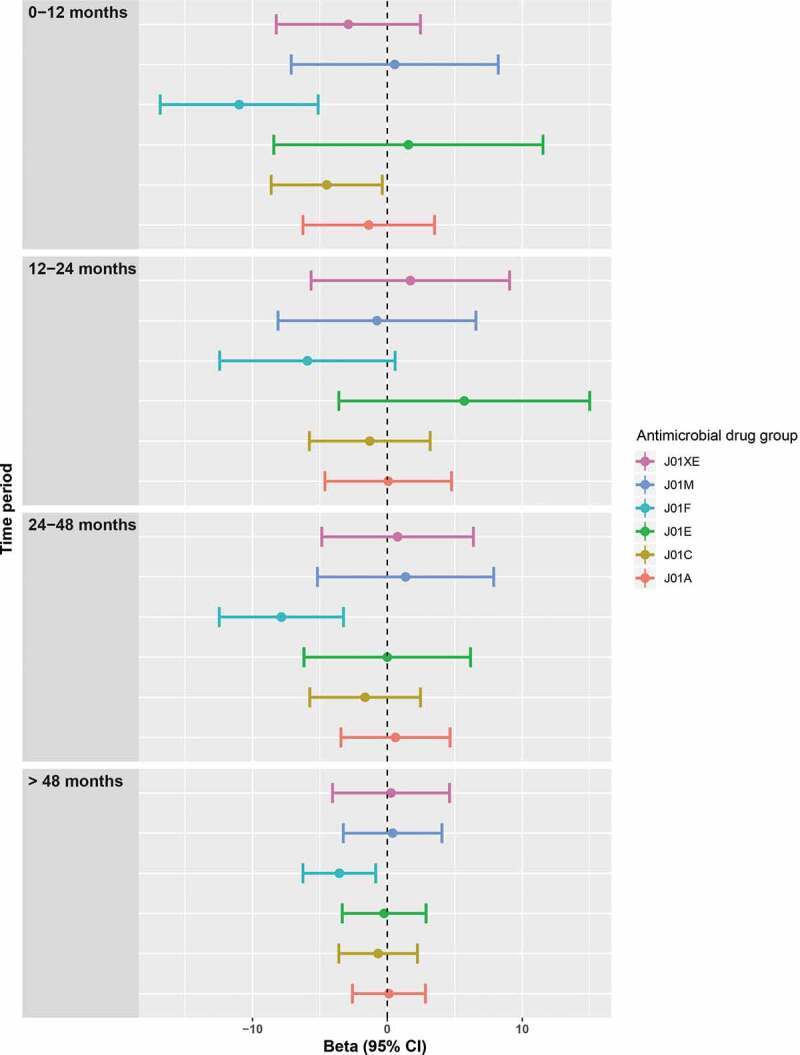
Diversity after antimicrobial drug use. Plots of the beta’s with 95% confidence intervals of the linear regression with as dependent variable the transformed (cube) Shannon alpha-diversity and as independent variables the different antimicrobial drug groups. All antimicrobial drug groups were analyzed with dummy variables with categories of use of 0–12, 12–24, 24–48 and >48 months compared to no use before sampling. The analyses were adjusted for age, sex, BMI, diabetes, time in mail, batch number, use of statins, PPIs, SSRIs, antipsychotics and systemic corticosteroids and (categorized) use of all other antimicrobial drugs.

The median overall diversity was 4.10 (IQR 3.73–4.37). The strongest and most prolonged effect on diversity was seen in the group of the macrolides and lincosamides (J01F). Transforming the beta’s back to physiological values (for an average person) resulted in a significantly lower diversity of 0.48 for 0–12 months after use of macrolides and lincosamides (J01F); a lower diversity of 0.28 (which was not significant when adjusting for all other antimicrobial drug use) for 12–24 months after use, a significantly lower diversity of 0.35 for 24–48 months after use and a significantly lower diversity of 0.17 for 48 months or longer after use. We also showed a significantly lower diversity of 0.24 after the use of beta-lactam antibacterials (J01C) within 1 year before feces sampling. No change in diversity was seen after the use of tetracyclines (J01A), sulfonamides and trimethoprim (J01E), quinolones (J01M) and nitrofurans (J01XE). (untransformed beta’s of model 2 in Figure 1)

**Figure 2.**
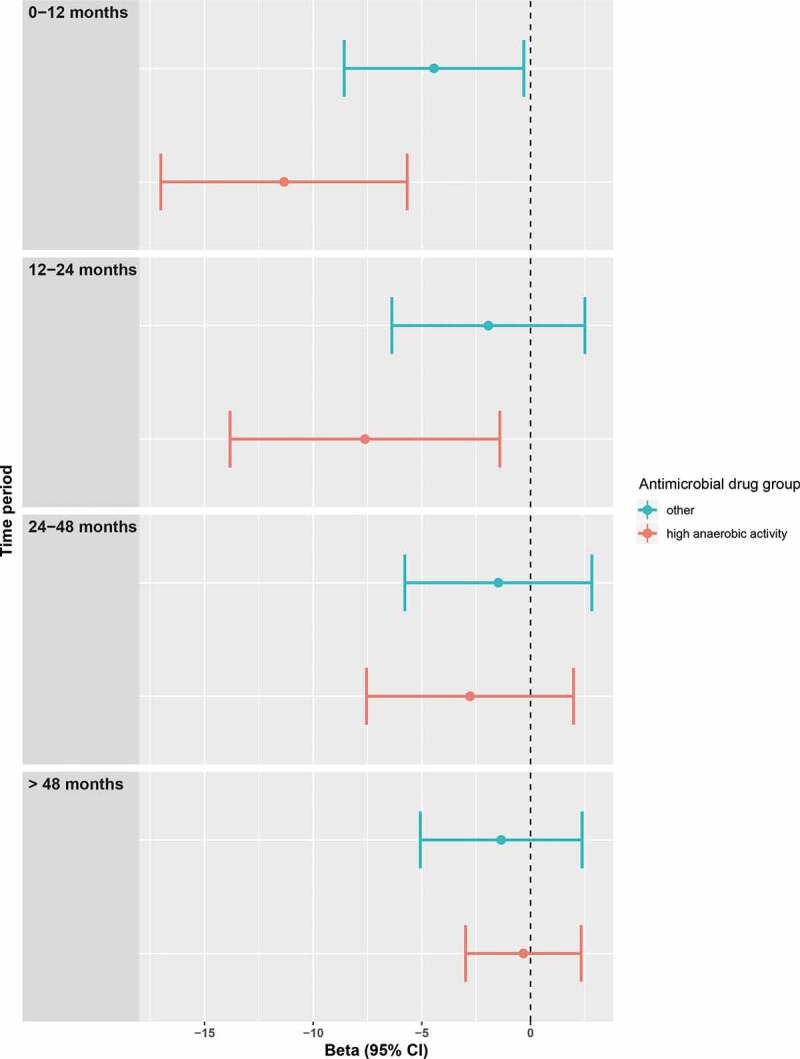
Diversity after using antimicrobial drugs with high anaerobic activity vs other antimicrobial drugs. Plots of the beta’s with 95% confidence intervals of the linear regression with as dependent variable the transformed (cube) Shannon alpha-diversity and as independent variables the combined antimicrobial drugs that have a strong anaerobic activity (anaerobic + drugs) and the combined remaining antimicrobial drugs (anaerobic- drugs). Both were analyzed with dummy variables with categories of 0–12, 12–24, 24–48 and >48 months compared to no use. The analyses were adjusted for age, sex, BMI, diabetes, time in mail, batch number, use of statins, PPIs, SSRIs, antipsychotics and systemic corticosteroids and (categorized) use of all other antimicrobial drugs.

We also classified the antimicrobial drugs in antimicrobial drugs with anaerobic activity (consisting of combinations of penicillins, including beta-lactamase inhibitors (J01CR), lincosamides (J01 FF) and imidazole derivatives (metronidazole) (J01XD): anaerobic+) and a group without this activity (all other antimicrobial drugs: anaerobic-). The use of anaerobic+ antimicrobial drugs was associated with a stronger and more prolonged effect on diversity than the use of antimicrobials without this activity. For an average person, diversity after the use of anaerobic+ antimicrobial drugs was 0.51 lower for the 0–12 months period and 0.36 lower for the 12–24 months period. Diversity was only 0.23 lower (for an average person) 0–12 months after the use of anaerobic-antimicrobial drugs. (untransformed beta’s in Figure 2) We also performed two sensitivity analyses with additional adjustment for diet and smoking, which slightly shifted the use of beta-lactams in the first year before sampling, resulting in a not significant difference. (Fig. S1 and S2)

### Firmicutes/Bacteroidetes ratio

**Figure 3.**
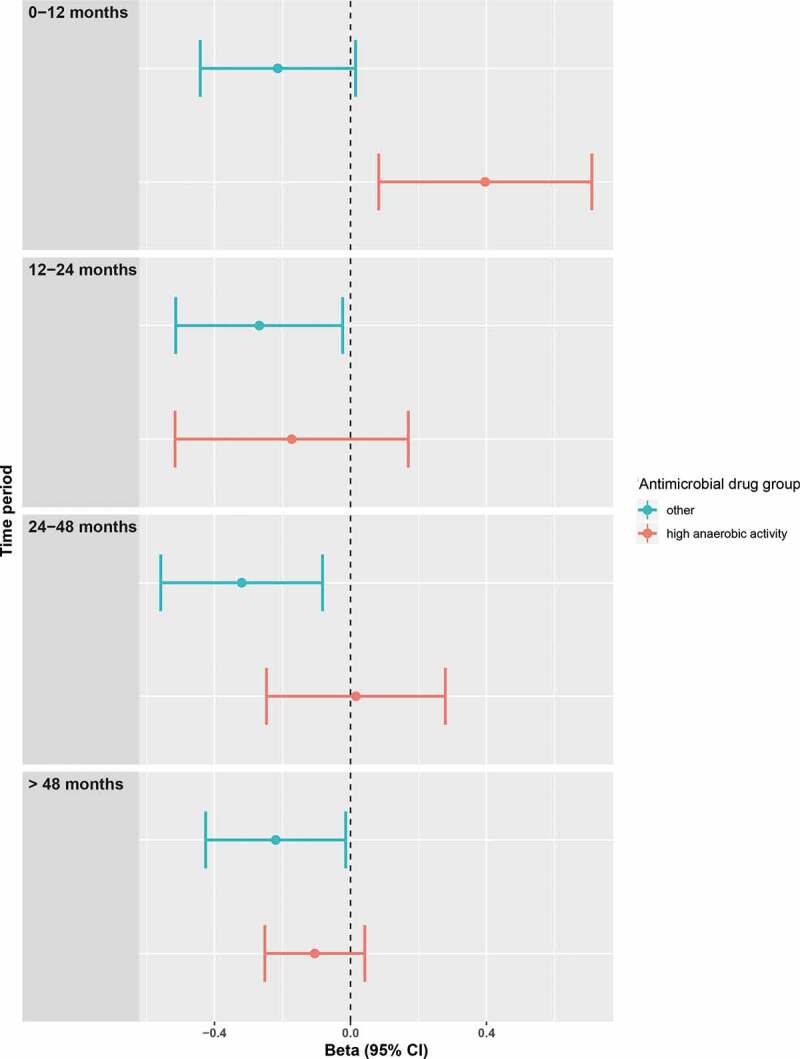
Firmicutes/Bacteroidetes ratio after using antimicrobial drugs with high anaerobic activity vs other antimicrobial drugs. Forest plots of the relative risks with 95% confidence intervals of the linear regression with as dependent variable the transformed (logarithmic) Firmicutes/Bacteroidetes ratio and as independent variables the combined antimicrobial drugs that have a strong anaerobic activity and the combined remaining antimicrobial drugs (“other”). Both were analyzed with dummy variables with categories of 0–12, 12–24, 24–48 and >48 months compared to no use. The analyses were adjusted for age, sex, BMI, diabetes, time in mail, batch number, use of statins, PPIs, SSRIs, antipsychotics and systemic corticosteroids and (categorized) use of all other antimicrobial drugs. A positive beta indicates a shift toward Firmicutes, whereas a negative beta indicates a shift toward Bacteroidetes.

The median F/B ratio was 0.085 (IQR 0.037–0.21). We could not show any significant differences for any of the different antimicrobial drug groups on the F/B ratio, both in model 1 and in model 2, in which we additionally adjusted for all antimicrobial drug use. (untransformed beta’s for model 2 in Figure S3). However, the F/B ratio significantly shifted toward Firmicutes in the 0–12 months before sampling after the use of anaerobic+ antimicrobial drugs. Furthermore, a significant shift could be demonstrated 12–24 months, 24–48 months and 48 months after the use of anaerobic- antimicrobial drugs toward Bacteroidetes of respectively 0.18, 0.20 and 0.16. (untransformed beta’s in Figure 3).

### Community structure

**Figure 4.**
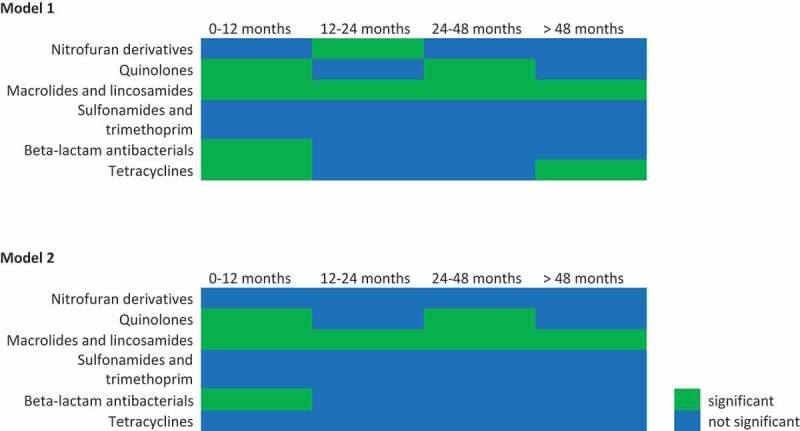
Effects of antimicrobial drug use on community structure. Significance table for all antimicrobial drug groups for all time categories studied in model 1, thus adjusted for age, sex, BMI, diabetes, time in mail, batch number, use of statins, PPIs, SSRIs, antipsychotics and systemic corticosteroids. (Top) Significance table for all antimicrobial drug groups for all time categories studied in model 2, thus adjusted for age, sex, BMI, diabetes, time in mail, batch number, use of statins, PPIs, SSRIs, antipsychotics and systemic corticosteroids and (categorized) use of all other antimicrobial drugs. (Bottom) In these significance tables green indicates significant (*p* < 0.05), blue indicates not significant.

Concerning the community structure (beta-diversity), we again found significant differences for macrolides and lincosamides (J01F) in all time categories. We also found a difference for beta-lactams (J01C) in 0–12 months before sampling. Finally, we found significant differences for quinolones both for 0–12 and 24–48 months before sampling, but not for 12–24 months (Figure 4). Most and strongest differences in genera were seen after the use of macrolides and lincosamides (Supplementary Table 2).

## Discussion

In this study, we showed an association between the use of different oral antimicrobial drugs and the diversity of the gut microbiota in feces samples of a middle-aged and elderly community-dwelling population. The strongest and most prolonged effects on the microbiota diversity were shown for macrolides and lincosamides, and these effects lasted up to several years after use. Also, diversity was lower and community structure was different in the first year after beta-lactam use. Furthermore, the use of antimicrobial drugs with a high anaerobic activity was associated with a shift toward Firmicutes. This, in contrary to the use of antimicrobial drugs without this activity, which resulted in s shift toward Bacteroidetes.

Increasing evidence shows that changes in the microbiota by antimicrobial drugs are associated with a variety of diseases. A study in Finnish school children reported an association between frequent macrolide use in early life (<2 years) and the development of asthma.^20^ Furthermore, the use of several antimicrobial drug groups was shown to be associated with several cancers in a large-nested case–control study, possibly acting via the gut microbiota.^21^ Also, but still unproven, it has been suggested to prescribe probiotics simultaneously with antimicrobial drugs. A large systemic review showed that there is evidence that probiotics are effective in preventing *Clostridium difficile*-associated diarrhea in not-immunocompromised individuals with a high baseline risk.^22^ Furthermore, in mice concurrent probiotics treatment during or after antibiotic therapy caused suppression of *Enterobacteriaceae* outgrowth, while promoting blooming of Firmicutes.^23^ Therefore, the influence on the gut microbiota should be taken into consideration when prescribing antibiotic drugs.

Many studies investigated the microbiota with different outcome parameters, included few participants or reported only short-term effects.^17–19^ A strength of our study is that we studied the gut microbiota in a large population-based cohort with detailed information on antimicrobial drug prescriptions and over more than a 4-year time period using different outcome parameters. Furthermore, we adjusted our models for several potential confounders, such as sex, age, BMI, diabetes and use of co-medication (statins, systemic corticosteroids, proton pump inhibitors, SSRIs and antipsychotics). Diversity is the most frequently reported outcome in microbiota studies and loss of diversity appears as the most consistent finding of intestinal dysbiosis.^24^ We also report on the Firmicutes/Bacteroidetes ratio, and the community structure, using the beta-diversity. The different outcome parameters enable a broader interpretation of the effect of antibiotic use on gut microbiota. Unfortunately, we could not compare the microbiota after with the microbiota before antimicrobial drug use, but the time frame, the cohort size and the fact that the participants were prescribed antibiotics by their physician for an infection and not specifically for this study made such a study design not feasible. Also, because of the length of the study time, other factors, such as intestinal surgery, could also have influenced the microbiota. However, because of the size of the cohort, we assume that the effect of these factors is small. Another limitation is that the feces was sent to the study center by the participants via mail, which might have influenced changes of the microbiota composition by environmental factors, such as temperature. However, the effects of our collection method have been studied and has resulted in the exclusion of samples that were in the mail longer than three days. Furthermore, the time in the mail was included as a covariate in the analyses. Also, the microbiota within our cohort had similar profiles as those in two other large population-based cohorts.^25^ Another limitation may be that our results were obtained from feces samples and may not reflect the microbiota more proximal in the digestive tract.^26^ Furthermore, we only used the last prescription before sampling, not taking into account the antimicrobial drug prescriptions used previously. However, we showed that correlations between the use of antimicrobial drugs of different classes were low and additionally, we adjusted for all other antimicrobial drug use.

All our results pointed to macrolides and lincosamides as the antimicrobial drugs with the highest ability to cause changes in the composition of the gut microbiota. Of these two types of antibiotics, lincosamides such as clindamycin probably have the strongest effect, since we also showed that antibiotics with a high anaerobic activity (which included clindamycin) had strong associations with the diversity of the microbiota. Another study has also shown long-lasting effects of clindamycin on the gut microbiota but only up to 2 years.^17^ Furthermore, the macrolide azithromycin was shown to have effects up to 6 months,^18^ and a shift of the gut microbiota at phylum level was found in Finnish children of 2–7 years old after macrolide use in the 2 years before sampling.^20^ Our data indicate that a shift in the composition of the gut microbiota persists for a longer time period.

Beta-lactam antibiotics caused a lower diversity and differences in community structure in the first year after use. Beta-lactams have been associated with effects on the composition of the gut microbiota in several small studies.^17^ Use of tetracyclines has been associated with a relative increase in the abundance of Bacteroidetes.^27^ Doxycycline use was shown to be associated with a lower diversity and a relative increase of Bacteroidetes in mice.^28^ Nitrofurantoin was shown to have only minor effects in a study in patients with urinary tract infections.^29^ These studies, however, investigated the effects after a maximum of a few months. Since we did not find any effects in our longer time periods, this might suggest that the effects of these antimicrobial drugs on the gut microbiota are restored after a few months. We also did not find any effects for sulfonamides and trimethoprim, but the use of these drugs (singly or in combination products) was very low.

Although we could not find an effect on the F/B ratio for separate antimicrobial drug classes, we found that the use of antimicrobial drugs with a high anaerobic activity was associated with a shift toward Firmicutes in the first year. In contrast, the use of antimicrobial drugs without this anaerobic activity was associated with a shift toward Bacteroidetes up to several years. Others also described effects on this ratio, but only directly after treatment, showing relatively more Bacteroidetes after the use of antimicrobial drugs.^30,31^

In conclusion, we showed that antimicrobial drugs, especially macrolides and lincosamides, are associated with a long-lasting shift in the gut microbiota. Further research is needed to explore the interaction and effect of specific antibiotics on the gut microbiota, considering the consequences of the use of antimicrobial drugs on the gut microbiota.

## Patients and methods

### Source population

The feces samples that were used in this study were obtained from study participants of the third cohort (RSIII) of The Rotterdam Study (RS), a prospective population-based study. This cohort includes 3122 individuals, who were recruited in the period March 2012 to June 2014 and who were 45 years and older, living in the Ommoord district in Rotterdam. All participants are invited every 3–4 years for follow-up interviews and examinations. More detailed information on the Rotterdam Study can be found elsewhere.^32^

### Gut microbiota composition

Stool samples were collected at home by the participants using a Commode Specimen Collection System (Covidien, Mansfield, MA). An aliquot of approximately 1 g was transferred to a 25 × 76 mm feces collection tube (Minigrip Nederland, Lelystad, The Netherlands) and sent through regular mail to the Erasmus MC. A short questionnaire addressing amongst others date and time of defecation was filled out by the participants (response percentage 69%). After receipt, the samples were stored at −20°C. Approximately, 300 mg of feces was homogenized in stool stabilizing buffer. Automated DNA-isolation (Arrow DNA; DiaSorin S.p.A., Saluggia, Italy) was performed using the Arrow DNA kit according to the manufacturer’s instructions and included bead-beating in Lysing Matrix B tubes containing 0.1 mm silica beads (MP Biomedicals, LLC, Bio Connect Life Sciences, Huissen, The Netherlands). The hypervariable regions V3 and V4 of the (bacterial) 16 S rRNA gene were amplified and sequenced using the Illumina MiSeq 2 × 300 base pairs protocol (FADROSH, PMID: 24558975). Phylogenetic multi-sample profiling was performed using an in-house developed pipeline based on the QIIME 1.9.0 (Caporaso PMID: 20383131) and USEARCH version 8.1 (Edgar PMID: 23955772) software packages. After subsampling at 10,000 reads per sample, taxonomy was assigned using the naïve Bayesian RDP classifier (vs 2.12)^33^ and the SILVA database (v128; Quast PMID: 23193283). The OTU table was cleaned by filtering out low abundance OTUs (<0.005% of total reads per OTUs and OTUs present in <1% of the samples). Samples with unknown information of time in the mail, samples arriving 3 days after collection and samples from participants who used antibiotics during or just before sampling were removed.

### Medication use

The date of the last prescription of an antimicrobial drug before feces sampling was obtained from a collaborative database of all community pharmacies in the Ommoord area that goes back to 1 January 1995. The antimicrobial drug prescriptions were grouped on Anatomical Therapeutic Chemical (ATC) code, which included: tetracyclines (J01A), amphenicols (J01B), beta-lactam antibacterials (J01C), other beta-lactam antibacterials (J01D) (which includes all generations of cephalosporins and carbapenems), sulfonamides and trimethoprim (J01E), macrolides and lincosamides (J01F) (J01F also includes streptogramins, but these were not prescribed in the study period), aminoglycoside antibacterials (J01G), quinolone antibacterials (J01M), glycopeptide antibacterials (J01XA), polymyxins (J01XB), steroid antibacterials (J01XC), imidazole derivatives (J01XD), nitrofuran derivatives (J01XE) and other antibacterials (J01XX). For each antimicrobial drug group, the time interval between the date of the last prescription and feces sampling was calculated and categorized into the use of 0–12, 12–24, 24–48 and >48 months before sampling or no use of the antimicrobial drug group. Additionally, the antimicrobial drugs were classified in a group antimicrobial drugs with a high activity against anaerobic species (anaerobic+), consisting of combinations of penicillins, including beta-lactamase inhibitors (J01CR), lincosamides (J01FF) and imidazole derivatives (metronidazole) (J01XD) and a group without this activity (anaerobic-) (all other antimicrobial drugs).

### Confounders

The following potential confounders (at the time of feces sampling) were taken into account in the analyses: age, sex, BMI, diabetes (use of anti-diabetic drugs (A10)), use of co-medication, time in the mail of the feces sample and batch number representing two batches of DNA isolation: the first 102 DNA isolation runs with a relatively high yield were labeled 0 and the last 32 runs with a relatively low yield were labeled 1. Patients who were prescribed a drug within 90 days before feces sampling were considered as current user of that drug. Drugs that possibly influence the composition of the microbiota according to literature: proton pump inhibitors (A02BC),^10^ statins (C10AA),^12^ systemic corticosteroids (H02),^11^ antipsychotics (N05A),^34^ selective serotonin reuptake inhibitors (SSRIs) (N06AB),^35^ antineoplastic agents (L01),^36^ and tacrolimus (L04AD).^37^ Since proton pump inhibitors may have been sold over the counter, participants were also asked if they used proton pump inhibitors.

Other potential confounders for studying the association with the gut microbiota are diet and smoking. Adjustment for diet was performed by adjusting for the dietary guidelines score (DGS), which is a score that varies from 0 to 14 and represents the adherence to the Dutch dietary guidelines which include 14 items: vegetables (≥200 g/day), fruit (≥200 g/day), whole-grains (≥90 g/day), legumes (≥135 g/week), nuts (≥15 g/day), dairy (≥350 g/day), fish (≥100 g/week), tea (≥450 mL/day), ratio whole-grains:total grains (≥50%), ratio unsaturated fats and oils:total fats (≥50%), red and processed meat (<300 g/week), sugar-containing beverages (≤150 mL/day), alcohol (≤10 g/day) and salt (≤6 g/day).^38^ Adjustment for smoking was performed by adjusting for the smoking status (never, ever, current).

### Statistical analyses

We performed several analyses in order to study the association between antimicrobial drug use and the composition of the gut microbiota, using several measures described below. For the diversity, Firmicutes/Bacteroidetes ratio (F/B ratio) and community structure analysis, we performed two models. In model 1, we adjusted for the above-mentioned confounders (sex, age, BMI, diabetes, use of co-medication, time in the mail and batch number). In model 2, we additionally adjusted for the categorized use of other antimicrobial drug groups (thus, for example: the association between tetracyclines and the gut microbiota was adjusted for the mentioned confounders and for categorized use of beta-lactam antibacterials, categorized use of sulfonamides and trimethoprim, categorized use of macrolides and lincosamides, categorized use of quinolones, and categorized use of nitrofuran derivatives). For diet and smoking, the data were not available for all participants: 269 (19.0%) were missing for diet and 108 (7.6%) for smoking. Therefore, adjustment for these confounders was only performed in two sensitivity analyses.

#### Diversity analysis

The Shannon index was used to calculate the alpha-diversity (measure of diversity of species within a sample). In order to obtain a normal distribution (according to the Kolgomorov–Smirnov test), it was transformed by calculating the cube. For each antimicrobial drug group, a linear regression was performed with the transformed Shannon alpha-diversity as the dependent variable, and four dummies for antimicrobial drug use of the specific group and the confounding variables as independent variables. The outcome was back transformed for male gender, batch 0, with median age, median BMI, for those who had no diabetes, who used no co-medication, of whom the sample was a median time in the mail and who used no other antimicrobial drugs than the one of interest (= average person). *P*-values <0.05 were considered to be significant.

#### The Firmicutes/Bacteroidetes ratio

The F/B ratio was calculated and logarithmically transformed to obtain a normal distribution. Different linear regressions were performed and transformed back to the average person as described above but now with as a dependent variable the transformed F/B ratio. *P*-values <0.05 were considered to be statistically significant.

#### Community structure analysis

MiRKAT is a recently developed package, available in the statistical program R, that tests for associations between microbiota composition and an outcome, using a semi-parametric kernel machine regression.^39^ MiRKAT (using 100,000 permutations) was used to investigate differences in the composition of the fecal microbiota using the Bray–Curtis beta-diversity distance (measure of dissimilarity of species composition between sample pairs). *P*-values <0.05 were considered to be statistically significant.

#### Single genera analyses

The genera that were significantly different in individuals who had used antimicrobial drugs were determined using the MaAsLin (Multivariate Association with Linear Models) function.^40^ The categorized variable for the use of each antimicrobial drug group was linearly included in the model (0 for no prescription at all, 4 for latest prescription in 0–12 months before sampling). For the analysis, the default settings were used: a false discovery rate of 25% and q-values <0.05 were considered to be statistically significant.

## Supplementary Material

Supplemental Material

## Data Availability

The datasets generated and/or analyzed during the current study are not publicly available due to privacy agreements of the participants of the study but are available from the corresponding author on reasonable request.
