# Supplementary material for: Long-term effects of antimicrobial drugs on the composition of the human gut microbiota
Source: Gut Microbes. 2020 Sep 29;12(1):1791677. doi: 10.1080/19490976.2020.1791677 (PMC7781642; doi:10.1080/19490976.2020.1791677)
Supplement: Supplemental Material [file KGMI_A_1791677_SM3408.zip › Supplementary information/Supplementary tables.docx]

**Table S1: Spearman’s coefficients for the antimicrobial drug groups**

|  | **J01A** | **J01C** | **J01E** | **J01F** | **J01M** | **J01XE** |
| --- | --- | --- | --- | --- | --- | --- |
| **J01A** | - | 0.23 | 0.11 | 0.31 | 0.14 | 0.06 |
| **J01C** | 0.23 | - | 0.11 | 0.18 | 0.17 | 0.11 |
| **J01E** | 0.11 | 0.11 | - | 0.16 | 0.32 | 0.38 |
| **J01F** | 0.31 | 0.18 | 0.16 | - | 0.14 | 0,15 |
| **J01M** | 0.14 | 0.17 | 0.32 | 0.14 | - | 0.26 |
| **J01XE** | 0.06 | 0.11 | 0.38 | -0.15 | 0.26 | - |

Table S1: Spearman’s coefficients for the different antibacterial drug groups. Correlations for each drug group were determined on the time interval between the last prescription of the drug group and the date of sampling the faces. J01A are tetracyclines; J01C are beta-lactam antibacterials; J01E are sulfonamides and trimethoprim; J01F are macrolides; lincosamides and streptogramins; J01M are quinolone antibacterials and J01XE are nitrofuran derivatives. Coefficients >0.8 are regarded as strong correlations.

**Figure S1: Diversity after antimicrobial drug use, sensitivity analysis with additional adjustment for diet.** Plots of the beta’s with 95% confidence intervals of the linear regression with as dependent variable the transformed (cube) Shannon alpha-diversity and as independent variables the different antimicrobial drug groups. All antimicrobial drug groups were analysed with dummy variables with categories of 0-12, 12-24, 24-48 and >48 months. The analyses were adjusted for age, sex, BMI, diabetes, time in mail, batch number and use of statins, PPIs, SSRIs, antipsychotics and systemic corticosteroids, (categorized) use of all other antimicrobial drugs and in addition for the dietary guideline score (DGS).

**Figure S2: Diversity after antimicrobial drug use, sensitivity analysis with additional adjustment for smoking status.** Plots of the beta’s with 95% confidence intervals of the linear regression with as dependent variable the transformed (cube) Shannon alpha-diversity and as independent variables the different antimicrobial drug groups. All antimicrobial drug groups were analysed with dummy variables with categories of 0-12, 12-24, 24-48 and >48 months. The analyses were adjusted for age, sex, BMI, diabetes, time in mail, batch number and use of statins, PPIs, SSRIs, antipsychotics and systemic corticosteroids, (categorized) use of all other antimicrobial drugs and in addition for smoking status (never, past, current).

**Figure S3: Firmicutes/Bacteroidetes ratio after antimicrobial drug use.** Plots of the beta’s with 95% confidence intervals of the linear regression with as dependent variable the transformed (logarithmic) Firmicutes/Bacteroidetes ratio and as independent variables the different antimicrobial drug groups. All antimicrobial drug groups were analysed with dummy variables with categories of 0-12, 12-24, 24-48 and >48 months. The analyses were adjusted for age, sex, BMI, diabetes, time in mail, batch number, use of statins, PPIs, SSRIs, antipsychotics and systemic corticosteroids and (categorized) use of all other antimicrobial drugs. **A positive beta indicates a shift towards Firmicutes, whereas a negative beta indicates a shift towards Bacteroidetes.**

**Table S2: Genera that were significantly lower or higher after the use of antimicrobial drugs.**

| **Lower after the use of tetracyclines** | |  |  |  |  |  |
| --- | --- | --- | --- | --- | --- | --- |
| *Phlyum* | *Class* | *Order* | *Family* | *Genus* | *N not 0* | *Estimate* |
| Firmicutes | Erysipelotrichia | Erysipelotrichales | Erysipelotrichaceae | Erysipelotrichaceae UCG003 | 1179 | -0,0034 |
| Firmicutes | Clostridia | Clostridiales | Lachnospiraceae | Tyzzerella | 374 | -0,0015 |
|  |  |  |  |  |  |  |
| **Lower after the use of beta-lactam antibacterials** | | |  |  |  |  |
| *Phlyum* | *Class* | *Order* | *Family* | *Genus* | *N not 0* | *Estimate* |
| Firmicutes | Clostridia | Clostridiales | Lachnospiraceae | gauvreauii group | 1188 | -0,0051 |
| Firmicutes | Clostridia | Clostridiales | Ruminococcaceae | Ruminococcaceae UCG010 | 1030 | -0,0021 |
| Firmicutes | Clostridia | Clostridiales | Lachnospiraceae | Lachnospiraceae FCS020group | 1301 | -0,0018 |
| Firmicutes | Clostridia | Clostridiales | Ruminococcaceae | Ruminococcaceae UCG003 | 986 | -0,0017 |
| Firmicutes | Clostridia | Clostridiales | Lachnospiraceae | Pseudobutyrivibrio | 430 | -0,0008 |
| Firmicutes | Clostridia | Clostridiales | Lachnospiraceae | Acetitomaculum | 271 | -0,0007 |
|  |  |  |  |  |  |  |
| **Higher after the use of beta-lactam antibacterials** | | |  |  |  |  |
| *Phlyum* | *Class* | *Order* | *Family* | *Genus* | *N not 0* | *Estimate* |
| Firmicutes | Clostridia | Clostridiales | Lachnospiraceae | Sellimonas | 435 | 0,0016 |
| Firmicutes | Erysipelotrichia | Erysipelotrichales | Erysipelotrichaceae | Erysipelatoclostridium | 811 | 0,0015 |
| Firmicutes | Clostridia | Clostridiales | Lachnospiraceae | gnavus group | 597 | 0,0011 |
| Firmicutes | Clostridia | Clostridiales | Ruminococcaceae | Oscillibacter | 1132 | 0,0011 |
| Firmicutes | Clostridia | Clostridiales | FamilyXIII | brachy group | 548 | 0,0008 |
| Firmicutes | Clostridia | Clostridiales | Ruminococcaceae | Anaerofilum | 1015 | 0,0008 |
| Firmicutes | Clostridia | Clostridiales | Lachnospiraceae | Eisenbergiella | 580 | 0,0007 |
| Firmicutes | Clostridia | Clostridiales | Lachnospiraceae | fissicatena group | 565 | 0,0006 |
| Firmicutes | Clostridia | Clostridiales | Lachnospiraceae | Hungatella | 476 | 0,0006 |
| Firmicutes | Erysipelotrichia | Erysipelotrichales | Erysipelotrichaceae | innocuum group | 384 | 0,0005 |
|  |  |  |  |  |  |  |
|  |  |  |  |  |  |  |
| **Higher after the use of sulfonamides and trimethoprim** | | |  |  |  |  |
| *Phlyum* | *Class* | *Order* | *Family* | *Genus* | *N not 0* | *Estimate* |
| Firmicutes | Clostridia | Clostridiales | Lachnospiraceae | Hungatella | 476 | 0,0017 |
|  |  |  |  |  |  |  |
|  |  |  |  |  |  |  |
| **Lower after the use of macrolides and lincosamides** | | |  |  |  |  |
| *Phlyum* | *Class* | *Order* | *Family* | *Genus* | *N not 0* | *Estimate* |
| Firmicutes | Clostridia | Clostridiales | Christensenellaceae | Christensenellaceae R7 group | 1352 | -0,0134 |
| Bacteroidetes | Bacteroidia | Bacteroidales | Prevotellaceae | Prevotella | 584 | -0,0125 |
| Firmicutes | Clostridia | Clostridiales | Ruminococcaceae | Ruminococcaceae UCG014 | 1180 | -0,0096 |
| Firmicutes | Clostridia | Clostridiales | Lachnospiraceae | Coprococcus | 885 | -0,0089 |
| Firmicutes | Clostridia | Clostridiales | Ruminococcaceae | Ruminococcaceae UCG002 | 1337 | -0,0085 |
| Bacteroidetes | Bacteroidia | Bacteroidales | Prevotellaceae | Paraprevotella | 644 | -0,0076 |
| Proteobacteria | Deltaproteobacteria | Desulfovibrionales | Desulfovibrionaceae | Desulfovibrio | 533 | -0,0070 |
| Firmicutes | Clostridia | Clostridiales | Ruminococcaceae | coprostanoligenes group | 1371 | -0,0057 |
| Bacteroidetes | Bacteroidia | Bacteroidales | Bacteroidales S247 group | unknown | 450 | -0,0051 |
| Actinobacteria | Coriobacteriia | Coriobacteriales | Coriobacteriaceae | Senegalimassilia | 794 | -0,0044 |
| Bacteroidetes | Bacteroidia | Bacteroidales | Prevotellaceae | Alloprevotella | 286 | -0,0041 |
| Firmicutes | Erysipelotrichia | Erysipelotrichales | Erysipelotrichaceae | Holdemanella | 660 | -0,0040 |
| Firmicutes | Clostridia | Clostridiales | Ruminococcaceae | Ruminococcaceae UCG005 | 1311 | -0,0038 |
| Firmicutes | Clostridia | Clostridiales | Ruminococcaceae | Ruminococcaceae UCG010 | 1030 | -0,0035 |
| Proteobacteria | Betaproteobacteria | Burkholderiales | Alcaligenaceae | Sutterella | 792 | -0,0031 |
| Firmicutes | Negativicutes | Selenomonadales | Veillonellaceae | Mitsuokella | 213 | -0,0028 |
| Bacteroidetes | Bacteroidia | Bacteroidales | unkown | unknown | 258 | -0,0023 |
| Actinobacteria | Coriobacteriia | Coriobacteriales | Coriobacteriaceae | Slackia | 666 | -0,0021 |
| Actinobacteria | Coriobacteriia | Coriobacteriales | Coriobacteriaceae | Enterorhabdus | 684 | -0,0021 |
| Firmicutes | Clostridia | Clostridiales | FamilyXIII | FamilyXIII AD3011 group | 1373 | -0,0020 |
| Firmicutes | Clostridia | Clostridiales | Ruminococcaceae | Ruminococcaceae UCG003 | 986 | -0,0020 |
| Firmicutes | Clostridia | Clostridiales | Ruminococcaceae | Intestinimonas | 1184 | -0,0019 |
| Actinobacteria | Coriobacteriia | Coriobacteriales | Coriobacteriaceae | Olsenella | 470 | -0,0017 |
| Firmicutes | Clostridia | Clostridiales | Lachnospiraceae | Lachnospiraceae AC2044 group | 589 | -0,0014 |
| Firmicutes | Clostridia | Clostridiales | Lachnospiraceae | Murimonas | 1108 | -0,0014 |
| Proteobacteria | Betaproteobacteria | Burkholderiales | Oxalobacteraceae | Oxalobacter | 440 | -0,0009 |
| Firmicutes | Clostridia | Clostridiales | Lachnospiraceae | oxidoreducens group | 567 | -0,0007 |
| Bacteroidetes | Bacteroidia | Bacteroidales | Porphyromonadaceae | unknown | 329 | -0,0006 |
|  |  |  |  |  |  |  |
| **Higher after the use of macrolides and lincosamides** | | |  |  |  |  |
| *Phlyum* | *Class* | *Order* | *Family* | *Genus* | *N not 0* | *Estimate* |
| Firmicutes | Bacilli | Lactobacillales | Streptococcaceae | Streptococcus | 1381 | 0,0067 |
| Firmicutes | Clostridia | Clostridiales | Ruminococcaceae | Ruminococcaceae UCG004 | 845 | 0,0025 |
| Firmicutes | Clostridia | Clostridiales | Lachnospiraceae | gnavus group | 597 | 0,0024 |
| Actinobacteria | Coriobacteriia | Coriobacteriales | Coriobacteriaceae | Eggerthella | 551 | 0,0024 |
| Firmicutes | Erysipelotrichia | Erysipelotrichales | Erysipelotrichaceae | Erysipelatoclostridium | 811 | 0,0023 |
| Actinobacteria | Coriobacteriia | Coriobacteriales | Coriobacteriaceae | Adlercreutzia | 888 | 0,0019 |
| Firmicutes | Clostridia | Clostridiales | Lachnospiraceae | Sellimonas | 435 | 0,0017 |
| Firmicutes | Clostridia | Clostridiales | Ruminococcaceae | Flavonifractor | 686 | 0,0013 |
| Firmicutes | Clostridia | Clostridiales | FamilyXIII | brachy group | 548 | 0,0010 |
| Actinobacteria | Coriobacteriia | Coriobacteriales | Coriobacteriaceae | Gordonibacter | 452 | 0,0007 |
| Firmicutes | Erysipelotrichia | Erysipelotrichales | Erysipelotrichaceae | Faecalitalea | 326 | 0,0006 |
| Firmicutes | Erysipelotrichia | Erysipelotrichales | Erysipelotrichaceae | innocuum group | 384 | 0,0005 |

*Table S2: Genera that were significantly different after the use of antimicrobial drugs. The analyses were performed using the MaAsLin package in R adjusting for age, sex, BMI, diabetes, time in mail, batch number, use of statins, PPIs, SSRIs, antipsychotics, systemic corticosteroids and the use of the other antimicrobial drug groups.*
