# Supplementary figures and images for: Long-term effects of antimicrobial drugs on the composition of the human gut microbiota
Source: Gut Microbes. 2020 Sep 29;12(1):1791677. doi: 10.1080/19490976.2020.1791677 (PMC7781642; doi:10.1080/19490976.2020.1791677)

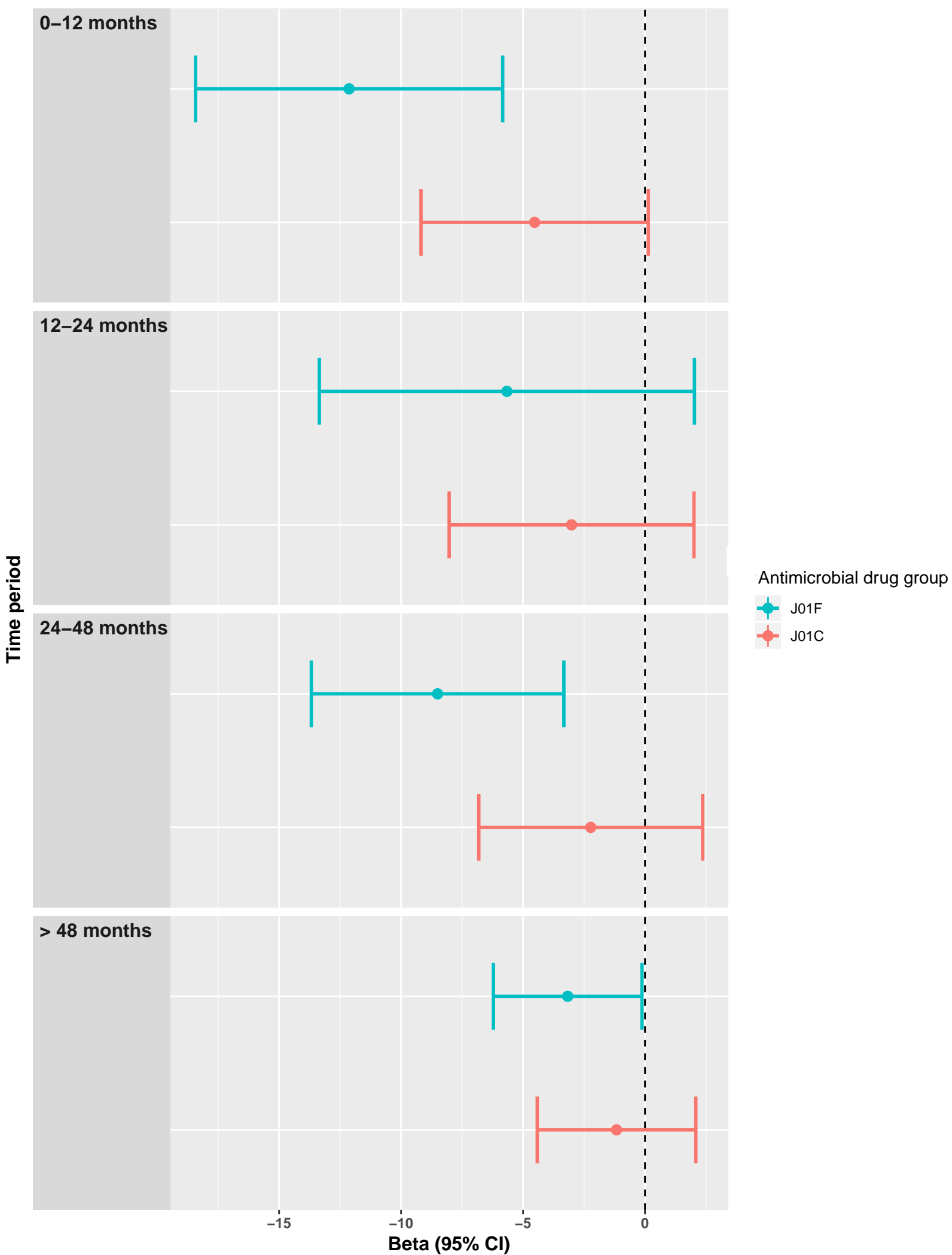

Supplement: Supplemental Material [file KGMI_A_1791677_SM3408.zip › Supplementary information/FigS1-Rplot_alpha_dieet.pdf]

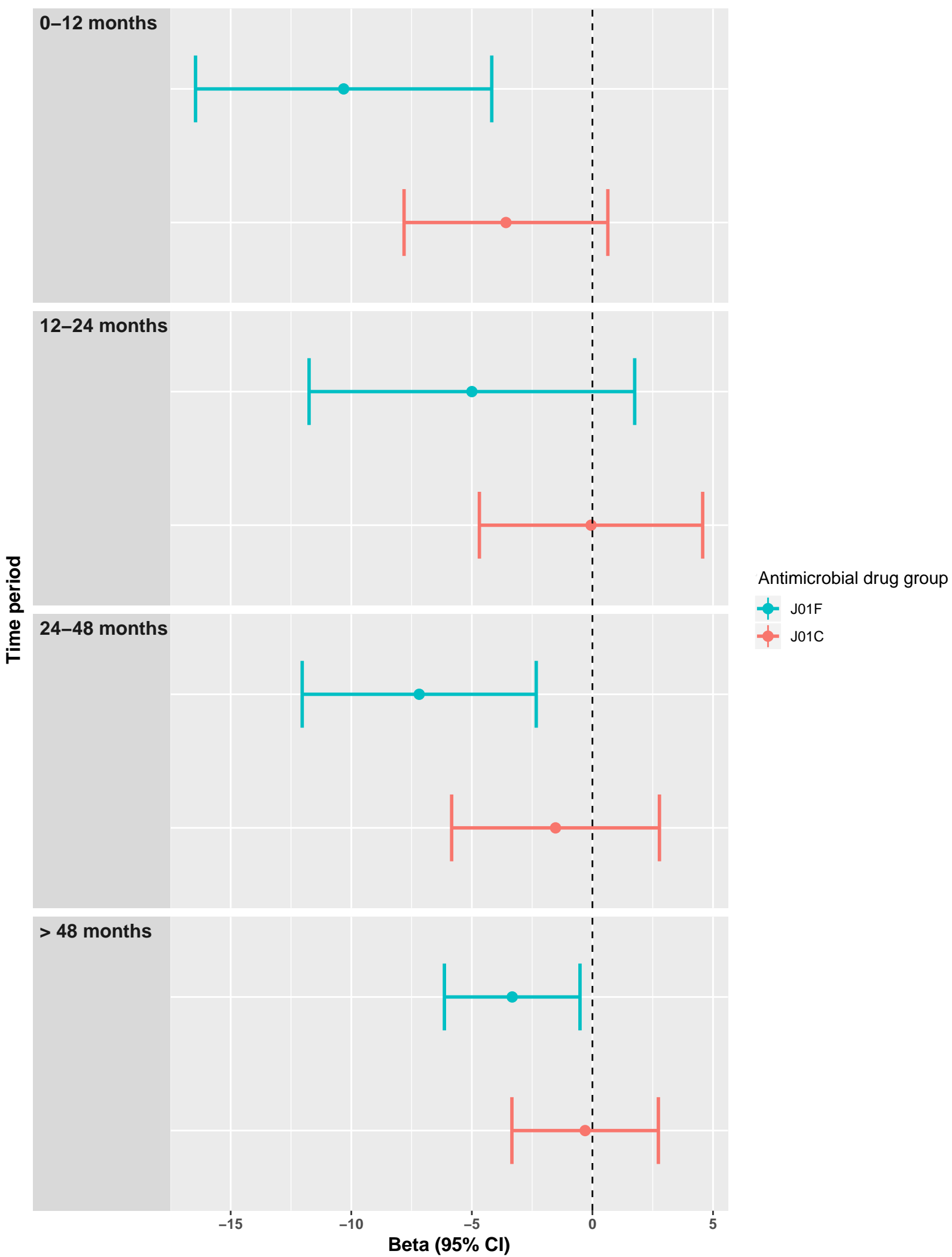

Supplement: Supplemental Material [file KGMI_A_1791677_SM3408.zip › Supplementary information/FigS2-Rplot_alpha_roken.pdf]

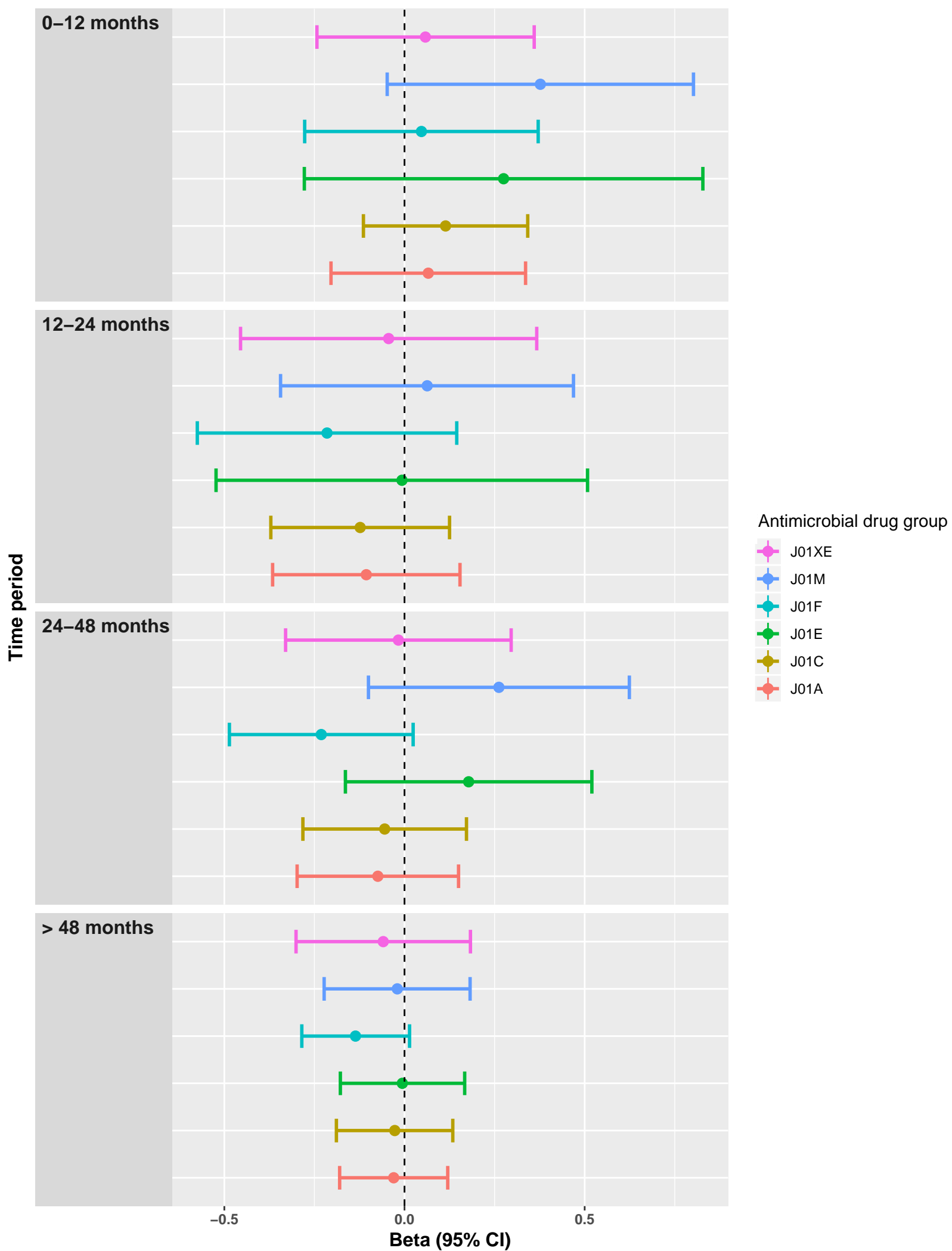

Supplement: Supplemental Material [file KGMI_A_1791677_SM3408.zip › Supplementary information/FigS3-Rplot_FB_corr.pdf]
